# Chestnut inner shell extract inhibits viral entry of porcine epidemic diarrhea virus and other coronaviruses *in vitro*

**DOI:** 10.3389/fvets.2022.930608

**Published:** 2022-09-02

**Authors:** Jinman Kim, Sohee Jo, Yeojin Choi, Tae-Won Kim, Jung-Eun Park

**Affiliations:** ^1^College of Veterinary Medicine, Chungnam National University, Daejeon, South Korea; ^2^Research Institute of Veterinary Medicine, Chungnam National University, Daejeon, South Korea

**Keywords:** chestnut inner shell, porcine epidemic diarrhea (PED), coronavirus, entry, inhibition

## Abstract

Porcine epidemic diarrhea virus (PEDV) is a coronavirus that causes acute diarrhea in suckling piglets. Although vaccines are able to reduce the incidence of PEDV infection, outbreaks of PEDV continue to be reported worldwide and cause serious economic losses in the swine industry. To identify novel antiviral sources, we identified the chestnut (*Castanea crenata*) inner shell (CIS) as a natural material with activity against PEDV infection *in vitro*. The ethanol fractions of CIS extracts potently inhibited PEDV infection with an IC90 of 30 μg/ml. Further investigation of the virus lifecycle demonstrated that CIS extract particularly targeted the early stages of PEDV infection by blocking viral attachment and membrane fusion at rates of 80~90%. In addition, CIS extract addition reduced the viral entry of other members of the *Coronaviridae* family. Our data demonstrated that CIS extract inhibited PEDV infection by blocking cell entry *in vitro* and suggest that CIS extract is a new prophylactic and therapeutic agent against PEDV and other coronavirus infections.

## Introduction

Porcine epidemic diarrhea virus (PEDV) infection is a highly contagious enteric disease associated with acute watery diarrhea, vomiting, and dehydration in suckling piglets (1–3). PEDV was first identified in 1978 in Europe (4) and then spread to Asian countries (5). A pathogenic variant of PEDV with higher morbidity and mortality emerged in China in 2010, and since then, this variant has been reported in many countries in Europe, Asia, and the United States and has caused considerable economic losses (6–8). Although commercial vaccines partially protect against the disease, PEDV endemic has persisted in many countries. Moreover, PEDV can persist in weaned piglets and sows with mild clinical symptoms (9, 10). Thus, novel effective prophylactics and/or therapeutics need to be developed.

Natural products are an essential source of novel chemical compounds with unique pharmacological activities. Among them, chestnut (*Castanea crenata*) inner shell extract (CISE) has benefits such as anti-wrinkling and whitening effects and anti-obesity, hepatoprotective, and antioxidant activities (11–17). These effects are considered to be mediated by the active components scopoletin and scoparone, but the content of these compounds in CISE is unknown (12, 14, 15, 17). Gallic acid and ellagic acid are the most abundant components in CISE (18, 19). The antiviral activity of gallic acid and ellagic acid has been shown for many viruses, such as human influenza virus A, human rhinovirus, Ebola virus, and human papilloma virus (20–23). These studies indicate the possibility that CISE may block viral infection, but the antiviral effect of CISE has not yet been reported.

In the present study, we first determined the antiviral activity of CISE on cells infected with PEDV. CISE inhibited viral entry by blocking virus–cell binding and membrane fusion and exhibited antiviral activity against other CoVs, which suggests that it has excellent potential as a natural broad-spectrum anti-coronavirus product.

## Materials and methods

### Cells

HEK293T (ATCC^®^ CRL-3216^TM^), Huh7 (KTCC KCLM60104), and Vero (ATCC^®^ CCL-81^TM^) cells were maintained in Dulbecco's modified Eagle's medium (DMEM) supplemented with 10% fetal bovine serum, 100 IU/ml penicillin, and 100 μg/ml streptomycin.

### Plasmids

Plasmids encoding pNL4.3-Luc.R-E-, vesicular stomatitis virus glycoprotein (VSV G), severe acute respiratory syndrome (SARS)-CoV spike (S) and Middle East respiratory syndrome (MERS)-CoV S were obtained from Tom Gallagher (Loyola University Chicago, Maywood, IL, USA). Plasmids encoding SARS-CoV-2 S with a C-terminal C9 tag were synthesized by GenScript and cloned into the pcDNA3.1 (+) vector (Invitrogen) using *EcoRI/NotI* sites.

### Virus propagation and titration

PEDV strain SM98 was propagated in Vero cells as described previously (24). Briefly, Vero cells were washed twice with phosphate-buffered saline (PBS) and inoculated with PEDV at a multiplicity of infection (MOI) of 0.01. After 2 h, the inoculums were removed, and the cells were incubated in DMEM containing 10 μg/ml trypsin (Sigma–Aldrich) for 24 h.

The PEDV titers were determined by plaque assay. Vero cells were inoculated with 10-fold serially diluted virus. After 1 h at 37°C, the inoculums were aspirated, and the cells were maintained in minimum essential medium containing 0.6% agar and 10 μg/ml trypsin. After 2 days, the cells were stained with a crystal violet solution (Sigma–Aldrich).

### Preparation of CISE

CISE was prepared according to a previous study (25). In brief, *Castanea crenata* (Shingongju Chestnut Agricultural Cooperative, Gong-ju, Korea) was peeled, and the chestnut inner shell was dried at 40°C for 3 days. The dried inner shell was ground to powder with a crusher, and the powder (300 g) was soaked in 70% ethanol (3 L) at room temperature for extraction. After 24 h of incubation, the aqueous layer was collected, and the extraction was repeated 3 times. The collected supernatant was concentrated with a rotary evaporator (Heidolph, Schwabach, Germany) at 40°C and freeze–dried with a HyperCOOL 3110 (Hanil Scientific Inc., Seoul, Korea). The lyophilized extract (40 g) was suspended in water (100 ml) and then subjected to conditioned Diaion HP-20 (Mitsubishi Chemical, Tokyo, Japan) open column (5 × 40 cm) chromatography. Elution was performed with distilled water (2 L) followed by ethanol (2 L). Each water and ethanol fraction was concentrated and freeze–dried, and the extraction yields for the water (CISE-DW) and ethanol (CISE-ET) fractions were 7.5 and 1.5%, respectively. The reproducibility of the CISE preparation was confirmed by additional extractions and HPLC analysis using raw materials from the same region and period.

### High-performance liquid chromatography analysis

Serial dilutions of the reference standards gallic acid and ellagic acid were prepared in methanol and 10% DMSO in methanol, respectively. The sample peaks were confirmed by comparing the reference standard peak in terms of the retention time (RT) and consistency using an ultraviolet (UV) detector. The analysis was performed by HPLC coupled with a UV detector (1,260 Infinity II LC System, Agilent), and the chromatographic separation assay was performed with an Eclipse Plus C18 analytical column (Agilent, 150 × 2.6 mm, 3.5-μm particle size) according to a previous study (25). The mobile phase consisted of 0.1% formic acid in distilled water (A) and acetonitrile (B), the flow rate was 0.12 ml/min, and the linear gradient program was the following: 0–30 min (95–70% A), 30–57 min (70–5% A), and 57–70 min (5–95% A). The injection volume was 2 μl, and the wavelength was set to 275 nm.

### Cell viability assay

Cell viability was determined by the MTT assay. A total of 1 × 10^4^ cells per well were seeded into 96-well plates and incubated with CISE, CISE-ET, and CISE-DW at various concentrations for 24 h. EX-cytox (Itsbio, Korea) was added to each well and incubated at 37°C for 1 h. The absorbance was measured using a microplate spectrophotometer at 450 nm.

### *In vitro* antiviral assay

Vero cells grown in 12-well plates were washed with PBS and infected with PEDV at an MOI of 0.1 in the presence of various doses of CISE, CISE-ET, and CISE-DW at 37°C. At 2 h post-infection (hpi), the inoculums were removed, and the cells were maintained in DMEM containing various doses of CISE. At 24 hpi, the medium was removed, and the cells were freeze–thawed in serum-free medium (SFM). The virus infectivity was presented as a percentage of the virus titer in the treated group compared with that of the DMSO control group, and the 90% inhibitory concentration (IC90) was calculated.

For the time-of-addition experiment, Vero cells grown in 4-well plates were washed with PBS and infected with PEDV at an MOI of 1 for 2 h at 37°C. At 2 hpi, the virus inoculums were removed, and 30 μg/ml CISE-ET was added at 0, 2, 4, and 6 hpi. At 10 hpi, the medium was removed, and the cells were freeze–thawed in SFM.

For the virus–cell binding experiment, Vero cells grown in 4-well plates were washed with PBS and infected with PEDV at an MOI of 1 with or without 30 μg/ml CISE-ET for 1 h at 4°C. At 1 hpi, the inoculums were removed, and the cells were maintained in SFM for 8 h.

For the cell pretreatment experiment, Vero cells grown in 4-well plates were washed with PBS and incubated with 30 μg/ml CISE-ET for 1 h at 37°C. After 1 h, the cells were washed with PBS and infected with PEDV at an MOI of 1 for 2 h at 37°C. At 2 hpi, the inoculums were removed, and the cells were maintained in SFM for 8 h.

For the virus pretreatment experiment, PEDV was incubated with or without 30 μg/ml CISE-ET for 1 h at 37°C. After 1 h, PEDV was diluted in SFM, and cells were infected with diluted PEDV at an MOI of 1 for 2 h at 37°C. At 2 hpi, the inoculums were removed, and the cells were maintained in SFM for 8 h.

For the virus–cell fusion experiment, Vero cells were infected with PEDV at an MOI of 1 for 1 h at 4°C. After 1 h, the cells were washed with PBS and treated with 30 μg/ml CISE-ET and/or 10 μg/ml trypsin for 2 h at 37°C. At 2 hpi, the inoculums were removed, and the cells were maintained in SFM for 8 h. In all the experiments, the virus titers were examined by plaque assay as described above.

### Generation and transduction of pseudotyped viruses

HEK293T cells were transfected with plasmids encoding MERS-CoV S, SARS-CoV S, SARS-CoV-2 S or VSV G along with pNL4.3-Luc.R-E-. At 48 h post-transfection, the supernatants were collected, filtered through 0.45-μm syringe filters, and stored at −80°C until use.

For transduction, Huh7 cells were transduced with pseudotyped viruses in the presence of various concentrations of CISE or CISE-ET for 1 h at 37°C. At 48 h post-transduction, the cells were lysed in cell culture lysis buffer (Promega), and the luciferase levels were measured by the addition of Fluc substrate (Promega) and the use of a Veritas microplate luminometer (Turner BioSystems). The virus infectivity is presented as a percentage of luciferase activity of the treated group compared with that of the DMSO control group, and the 50% inhibitory concentration (IC50) was calculated.

### Statistical analyses

All experiments were independently repeated at least two times. The data are presented as the means ± SDs. The statistical significance was calculated using the Holm–Sidak multiple Student's *t*-test procedure. A *P* < 0.05 was considered to indicate statistical significance.

## Results

### Preparation and characterization of CISE

**Figure 1:**
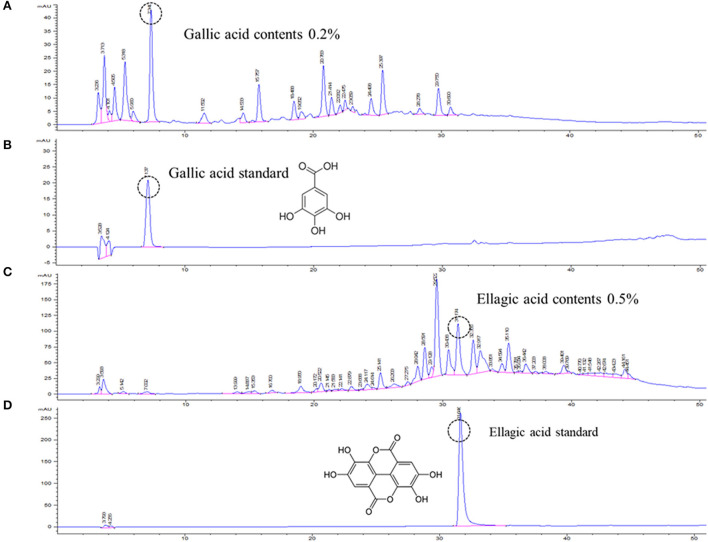
Representative chromatogram of CISE. Water fraction (CISE-DW, **A**) and ethanol fraction (CISE-ET, **C**) of CISE and the standard marker compounds gallic acid **(B)** and ellagic acid **(D)**.

Open column chromatography was performed to produce water and ethanol fractions, and a fingerprint chromatogram of the extract was obtained by HPLC analysis (Figure 1). Gallic acid and ellagic acid were analyzed as marker compounds of CISE-DW and CISE-ET, respectively. The linear regression analysis data for the standard compounds gallic acid and ellagic acid are provided in Table 1. CISE-DW was standardized with gallic acid, the content was ~0.2% of CISE-DW, CISE-ET was standardized with ellagic acid, and the content was ~0.5% of CISE-ET (Figure 1).

**Table 1:** Regression equation with the LODs and LOQs of gallic acid and ellagic acid.

**Compound**	**RT^a^ (min)**	**UV (nm)**	**Regression equation**	***r* ^2^ ^b^**	**LOD^c^ (μg/ml)**	**LOQ^d^ (μg/ml)**
Gallic acid	7.1	275	y = 42.3x + 3.6	0.999	0.15	0.5
Ellagic acid	31.7	275	Y = 36.6x – 21.6	0.999	0.15	0.5

a RT, retention time;

b r^2^, correlation coefficient;

c LOD, limit of detection;

d LOQ, limit of quantitation.

### CISE-ET inhibited PEDV infection *in vitro*

**Figure 2:**
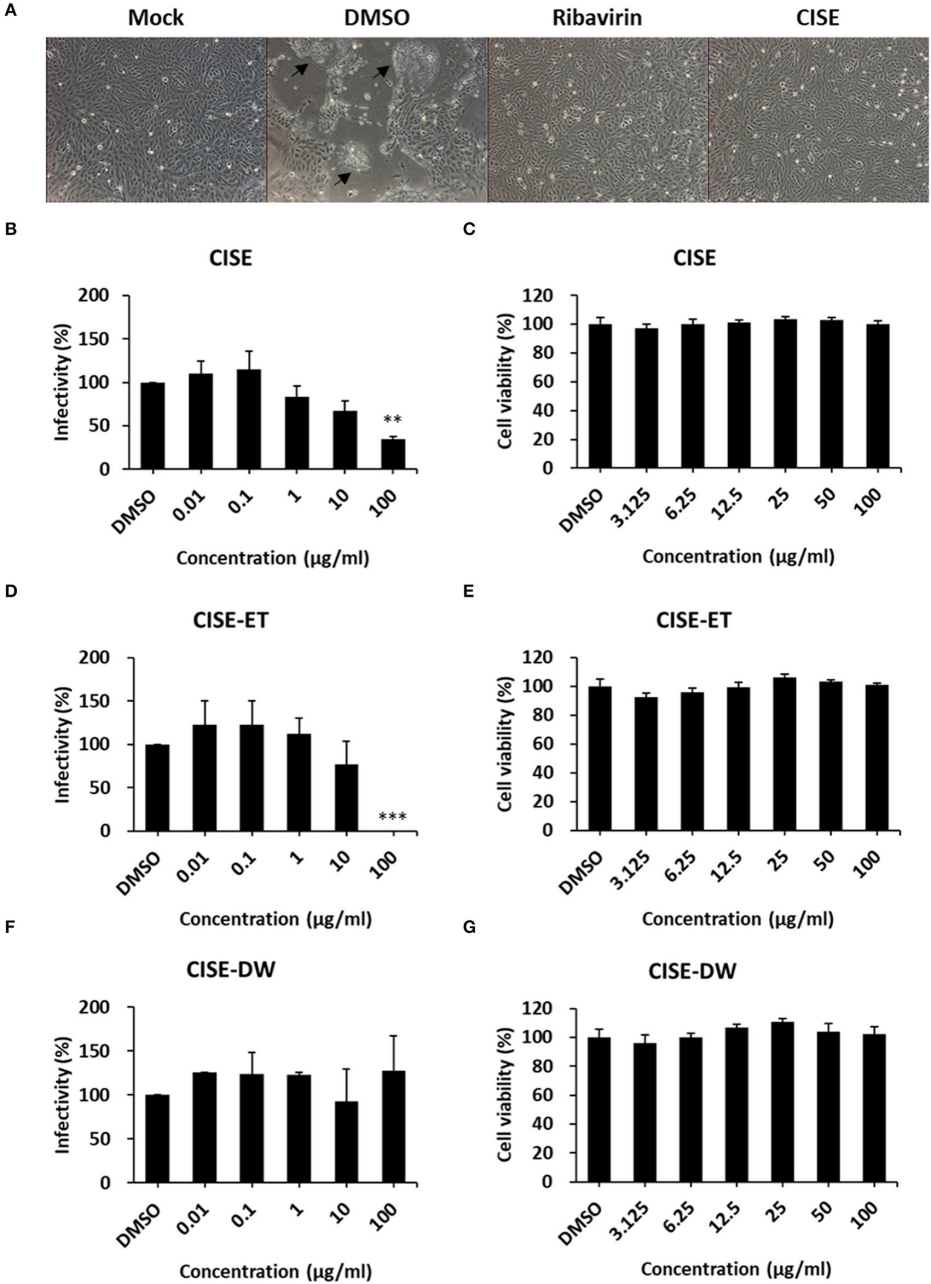
CISE inhibited PEDV infection in Vero cells. **(A)** Vero cells were infected with PEDV (MOI = 0.1) in the presence of CISE (50 μg/ml), ribavirin (50 μg/ml), or a DMSO control. At 24 hpi, a cytopathic effect (black arrow) was observed. **(B–G)** Vero cells were infected with PEDV (MOI = 0.1) in the presence of various concentrations of CISE **(B)**, CISE-ET **(D)**, or CISE-DW **(F)**. At 24 hpi, the virus titers were measured by plaque assay. Vero cells were incubated with various concentrations of CISE **(C)**, CISE-ET **(E)**, or CISE-DW **(G)** for 24 h, and the cell viability was then evaluated by the MTT assay. The antiviral activity and cell viability are expressed relative to those found for the DMSO control group. The error bars represent the SDs from the means (*n* = 3). **P < 0.01; ***P < 0.001.

To examine the PEDV antiviral activity of CISE, Vero cells were infected with PEDV in the presence or absence of CISE, and the cytopathic effect and virus titers were evaluated. As shown in Figure 2A, PEDV-infected cells in the DMSO control group showed a significant cytopathic effect (cell–cell fusion) at 24 hpi. The PEDV-associated cytopathic effect was greatly reduced in the presence of CISE and ribavirin, a broad-spectrum antiviral agent (Figure 2A). The virus titers were significantly decreased by CISE in a dose-dependent manner (Figure 2B, IC90 = 179 μg/ml) without cytotoxicity (Figure 2C).

To determine which component in the CISE exerts antiviral effects, CISE was further separated into water (CISE-DW) or ethanol (CISE-ET) fractions as described in Figure 1. Similar to CISE, CISE-ET inhibited PEDV infection in a dose-dependent manner (Figure 2D, IC90 = 30 μg/ml) without cytotoxicity (Figure 2E). Unlike CISE and CISE-ET, CISE-DW had no effect on PEDV infection or cell viability (Figures 2F,G). These data indicated that the components in CISE-ET exhibited antiviral activity *in vitro*.

### CISE-ET inhibited the early phase of PEDV infection

**Figure 3:**
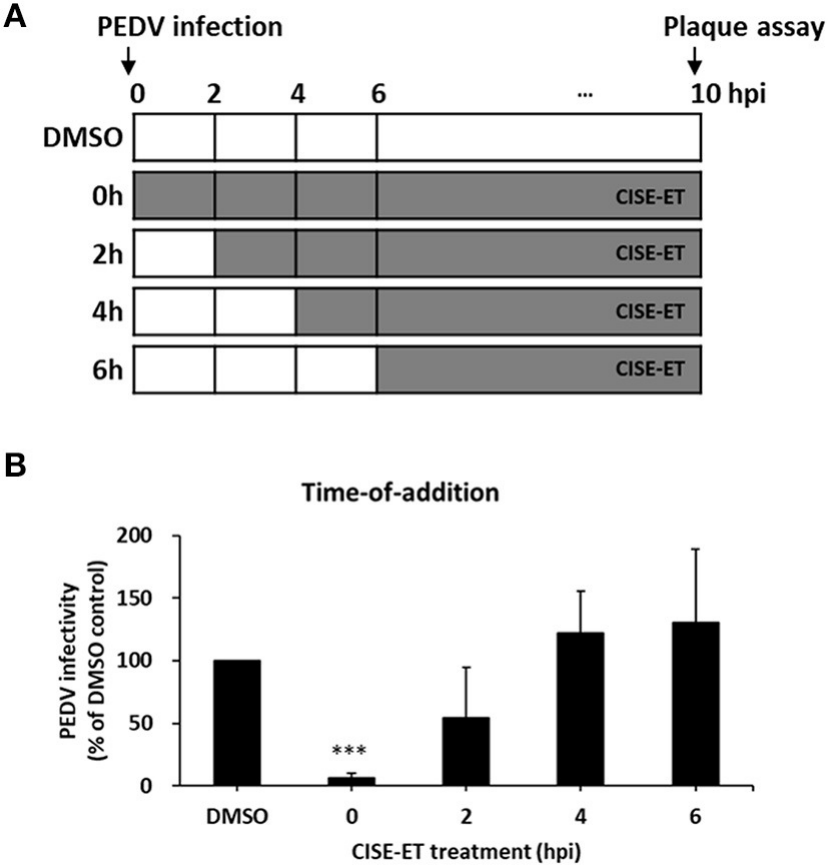
CISE-ET inhibited the early phase of PEDV infection. **(A)** Schematic of the time-of-addition experiments. Vero cells were infected with PEDV (MOI = 1), and 30 μg/ml CISE-ET was added at the indicated time points after virus infection. At 10 hpi, the virus titers were measured by plaque assay. **(B)** The viral infectivity is expressed relative to that obtained for the DMSO control group. The error bars represent the SDs from the means (*n* = 3). ***P < 0.001.

To determine the stage at which CISE-ET exerted inhibitory activity, we performed time-of-addition experiments with a concentration of 30 μg/ml (Figure 3A). More than 90% of viral infection was reduced by the administration of CISE-ET at the time of virus infection (0 h, Figure 3B, *P* < 0.001). No antiviral activity was observed when CISE-ET was added at or after 2 hpi (Figure 3B). These data indicated that CISE-ET inhibited the early phase of PEDV infection.

### CISE-ET inhibited virus–Cell binding by affecting the virus

**Figure 4:**
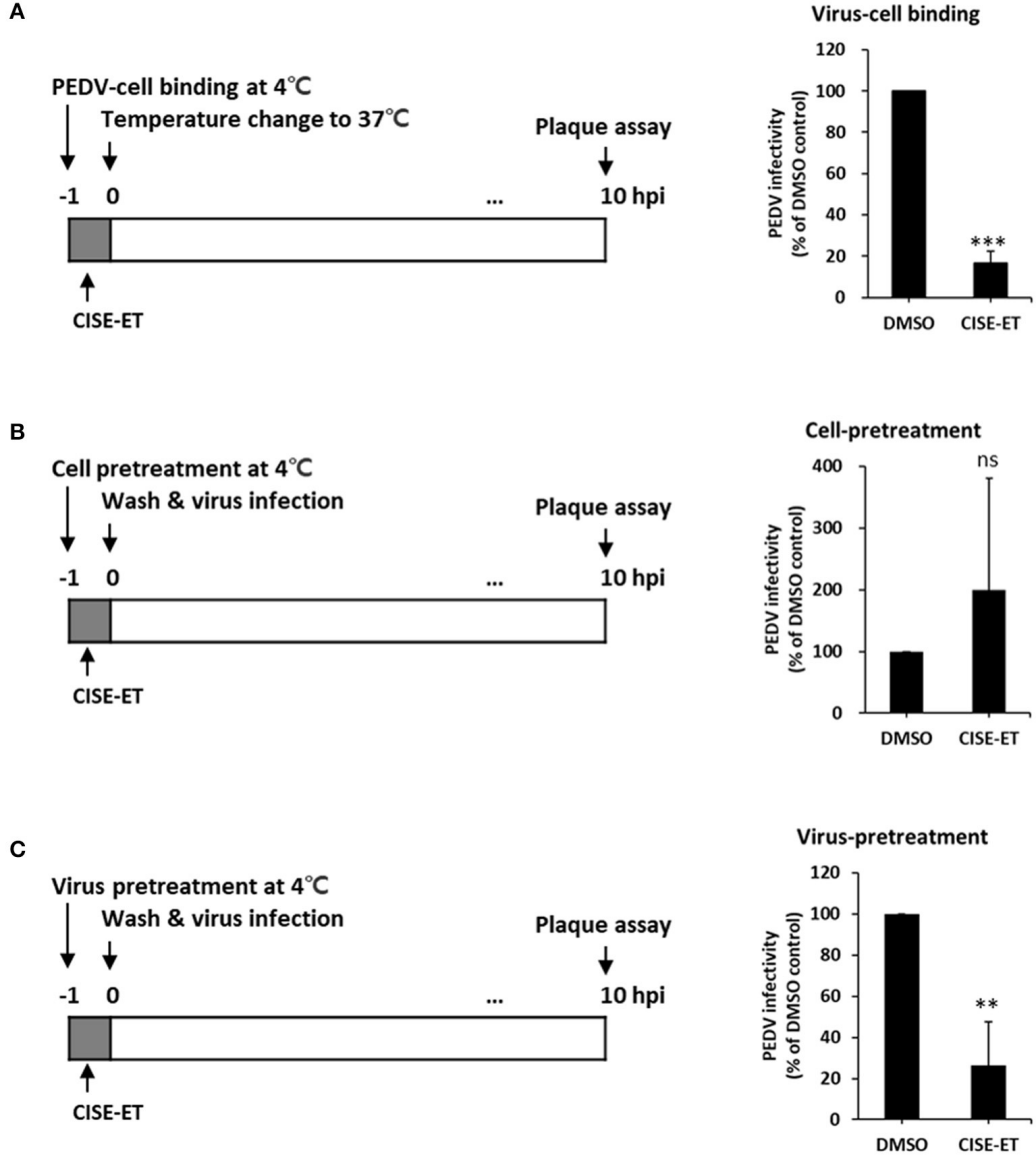
CISE-ET blocked virus–cell binding by inactivating PEDV particles. **(A)** Vero cells were infected with PEDV (MOI = 1), treated with 30 μg/ml CISE-ET at 4°C for 1 h, washed with PBS, and maintained in SFM for 10 h. **(B)** Vero cells were treated with 30 μg/ml CISE-ET at 4°C for 1 h, washed with PBS and infected with PEDV (MOI = 1) at 37°C. After 2 h, the cells were washed with PBS and maintained in SFM for 8 h. **(C)** PEDV was incubated with 30 μg/ml CISE-ET at 4°C for 1 h and then diluted in SFM 10-fold. Vero cells were then infected with diluted PEDV (MOI = 1) at 37°C for 1, washed with PBS and maintained in SFM for 8 h. At 10 hpi, the virus titers were measured by plaque assay. The viral infectivity is expressed relative to that found for the DMSO control group. The error bars represent the SDs from the means (*n* = 3). **P < 0.01; ***P < 0.001; ns, not significant.

CoV entry is mediated by the interaction of S protein with cellular receptors and subsequently by membrane fusion catalyzed by host proteases (26, 27). To examine whether CISE-ET inhibited virus–cell binding, we measured the antiviral activity of CISE-ET during virus–cell binding. CISE-ET treatment during virus–cell binding showed inhibition rates of ~80% (Figure 4A, *P* < 0.001). To further explore the mechanism through which CISE-ET inhibits virus–cell binding, we determined the antiviral activity of CISE-ET using two experiments: (i) cell pretreatment and (ii) virus pretreatment. No antiviral activity was observed when cells were pretreated with CISE-ET (Figure 4B, *P* = 0.3911). In contrast, virus pretreatment with CISE-ET resulted in inhibition rates of ~90% (Figure 4C, *P* < 0.001). These data indicated that CISE-ET inhibited virus–cell binding, most likely by inhibiting the initial processes of virus attachment and entry.

### CISE-ET inhibited virus–Cell fusion

**Figure 5:**
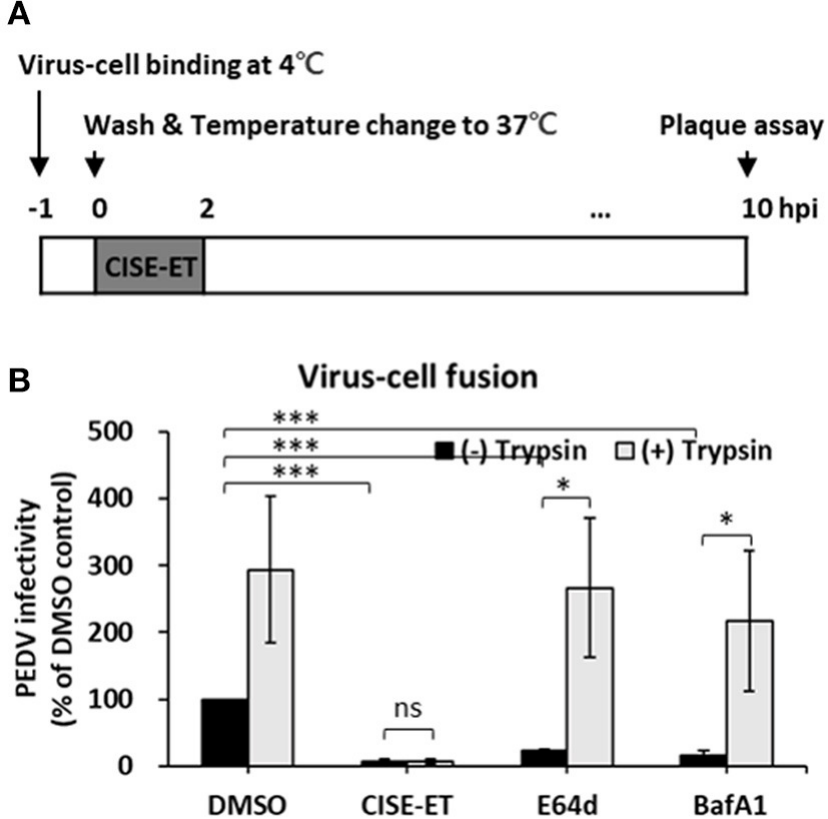
CISE-ET inhibited virus–cell fusion and proteolytic cleavage of S proteins. **(A)** Schematic of the synchronized infection experiments of virus–cell fusion. Vero cells were incubated with PEDV (MOI = 1) for 1 h at 4°C for initial binding, washed with PBS and treated with 30 μg/ml CISE-ET and/or 10 μg/ml trypsin for 2 h at 37°C. The inoculums were then removed, and the cells were maintained in SFM for 8 h. At 10 hpi, the virus titers were measured by plaque assay. **(B)** The viral infectivity is expressed relative to that found for the DMSO control group. The error bars represent the SDs from the means (*n* = 3). *P < 0.05; ***P < 0.001; ns, not significant.

We then examined whether CISE-ET blocks virus–cell fusion. As shown in Figure 5A, PEDV particles were allowed to first attach to the target cells at 4°C. After 1 h, unbound viruses were removed, and virus-bound cells were incubated at 37°C to initiate viral entry/fusion in the presence or absence of CISE-ET. In the absence of trypsin, the PEDV titers were significantly decreased under CISE-ET-treated conditions, similar to the results obtained with treatment with the endosomal cathepsin inhibitor E64d and treatment with the vacuolar H+ ATPase inhibitor BafA1 (Figure 5B, *P* < 0.001). These findings revealed that CISE-ET also strongly inhibited the post-attachment membrane fusion steps. The addition of exogenous trypsin increased PEDV infection in Vero cells by 10-fold and rescued PEDV infection under BafA1- and E64d-treated conditions (Figure 5B, *P* = 0.015, *P* = 0.030). Interestingly, enhancement of PEDV infection by trypsin was not observed in the presence of CISE-ET (Figure 5B, *P* = 0.731). These data indicated that CISE-ET inhibited PEDV S-mediated virus–cell fusion.

### CISE-ET inhibited the viral entry of MERS-CoV, SARS-CoV, and SARS-CoV-2

**Figure 6:**
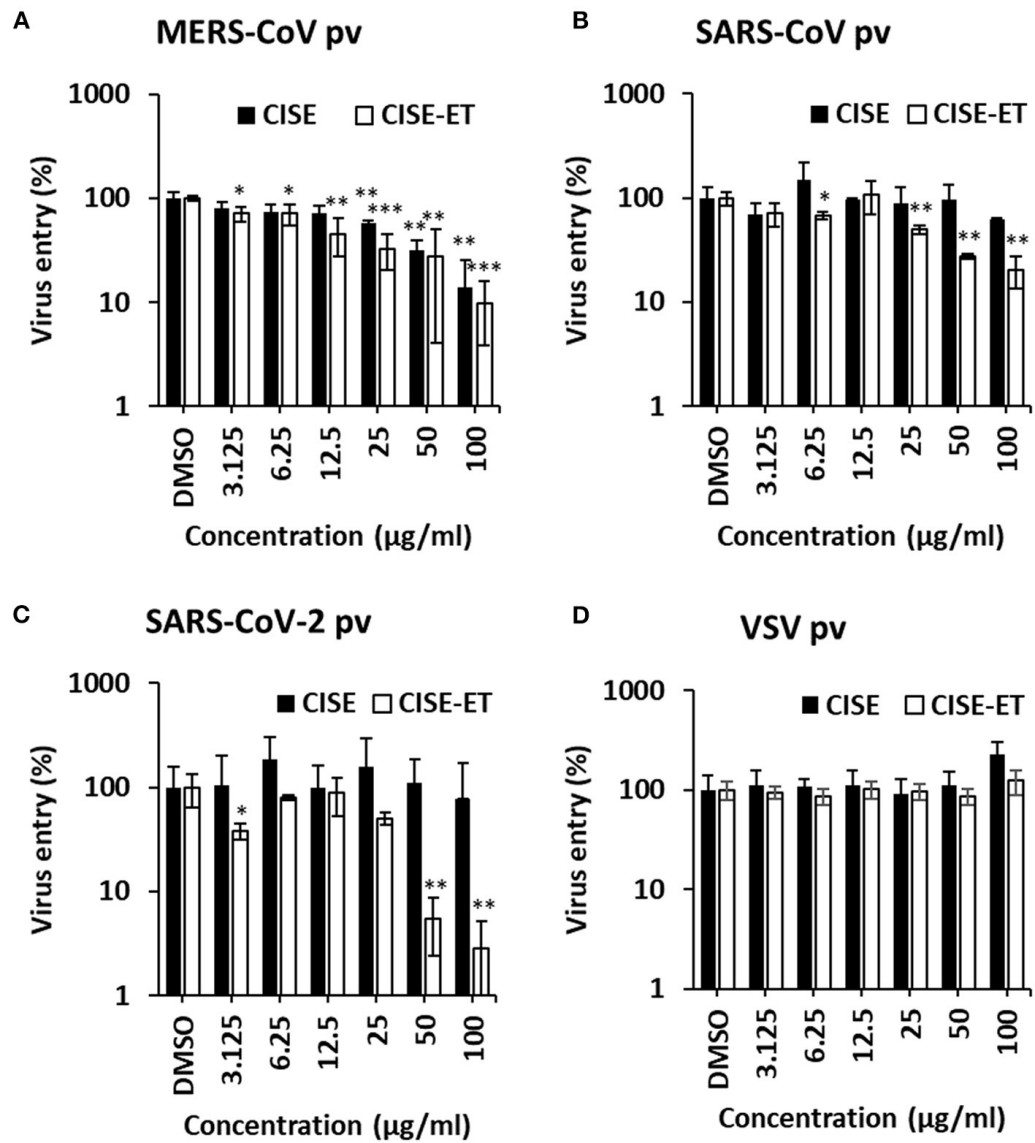
CISE-ET inhibited the entry of MERS-CoV, SARS-CoV and SARS-CoV-2. Huh7 cells were transduced with MERS-CoV **(A)**, SARS-CoV **(B)**, SARS-CoV-2 **(C)** or VSV **(D)** pseudotyped viruses (pvs) in the presence of various concentrations of CISE or CISE-ET. The viral entry was quantified by a luciferase assay at 48 h post-transduction and expressed relative to that found for the DMSO control group. The error bars represent the SDs from the means (*n* = 3). *P < 0.05; **P < 0.01; ***P < 0.001.

We subsequently sought to examine the effect of CISE on other viruses in the *Coronaviridae* family, including MERS-CoV, SARS-CoV, and SARS-CoV-2. Specifically, pseudotyped viruses expressing corresponding S proteins were generated and transduced into target cells in the presence of CISE or CISE-ET. The addition of CISE blocked the entry of MERS-CoV (Figure 6A, IC50 = 30 μg/ml) but did not affect SARS-CoV (Figure 6B) or SARS-CoV-2 (Figure 6C) pseudovirus transduction. CISE-ET treatment efficiently inhibited MERS-CoV (IC50 = 20 μg/ml, Figure 6A), SARS-CoV (IC50 = 14 μg/ml, Figure 6B), and SARS-CoV-2 (IC50 = 21 μg/ml, Figure 6C) pseudovirus transduction. VSV entry was not affected by either CISE or CISE-ET (Figure 6D). These results indicated that CISE also exhibited antiviral activity against select viruses in the *Coronaviridae* family.

## Discussion

There are currently no approved anti-PEDV therapeutics. The use of prophylactic/therapeutic treatment against PEDV may reduce the incidence of infection in endemic areas.

In an effort to develop antiviral drugs for PEDV, several naturally derived materials displaying anti-PEDV activity, including *Pogostemon cablin* polysaccharide (28), aloe (29), tomatidine (30), *Epimedium koreanum* Nakai (31), and various Vietnamese traditional medicinal plants (32), have been identified. In the present study, we demonstrated that CISE inhibits PEDV infection by targeting viral entry. CISE-ET blocked viral attachment and fusion to host cells without exerting any significant influence on viral replication or release. Our findings suggest that CIS is an important source of antiviral materials for the development of PEDV therapeutics.

Plant-derived extracts such as CISE contain numerous components, and their actions in cultured cells may be mutually exclusive. Moreover, the biological effect of natural compound extracts might be derived from the synergistic integration of multiple components with different potencies and efficacies. Gallic acid and ellagic acid are the most abundant components in CISE and exhibit antiviral activity against many viruses (20–23), and we thus speculate that these components might exert an antiviral effect. The analysis of the components in CISE has not been fully elucidated, and the identification of the leading active component is an essential step for drug development that can offer new therapeutic options for clinical use. We are currently preparing peak-by-peak fractions to identify the active ingredients in CISE-ET and will subsequently compare the potency of their antiviral activities.

The mode of action is an unanswered question. Our data provide evidence showing that CISE-ET specifically blocks virus entry by inhibiting PEDV attachment and post-attachment viral entry/fusion. CoV cell entry is orchestrated by S proteins that bind to cellular receptors and catalyze virus–cell membrane fusion (26, 33, 34). Studies have suggested that virus–cell membrane fusion is triggered by several activators of S proteins: (i) protease cleavage at S1/S2, (ii) receptor-induced conformational changes, (iii) protease cleavage at S2‘, and (iv) ionic changes (26, 35–37). Along with conformational changes in S proteins, curvature of the viral envelope and cellular membrane is needed for membrane fusion to occur (38, 39). Because CISE showed specific but broad antiviral activity against the tested CoVs (Figure 6), we initially hypothesized that CISE might exert its antiviral activity by inhibiting the proteolytic cleavage of S protein. However, the proteolytic processing of PEDV S and MERS-CoV S was not affected (data not shown). Alternatively, we speculate that CISE might apply its antiviral activity by intercalating with the surface structural components of PEDV, such as S glycoproteins and the viral envelope. For instance, the drug could interfere with conformational changes in S proteins and/or curvature of the viral envelope. Further analysis is necessary to clarify the specific molecular mechanism(s) of action.

## Conclusion

In conclusion, this study provides the first demonstration of the antiviral activity of CISE against PEDV and multiple CoVs. CISE impedes PEDV infection by blocking viral attachment and fusion. These findings suggest that CISE and its components could be developed as therapeutic or prophylactic agents for restricting the viral entry of selected CoVs.

## Data availability statement

The original contributions presented in the study are included in the article/supplementary material, further inquiries can be directed to the corresponding authors.

## Author contributions

J-EP and T-WK: conceptualized the study and prepared the manuscript and supervised the project and acquired the funding. J-EP, JK, SJ, YC, and T-WK: designed the study methodology and analyzed data. All the authors reviewed and edited the manuscript and have read and agreed to the published version of this manuscript.

## Funding

This work was supported by the National Research Foundation of Korea (NRF) grant funded by the Korean government (MSIT) (2020R1C1C1012854), the Basic Science Research Program through the National Research Foundation of Korea funded by the Ministry of Education (2021R1A6A1A03045495), and the research fund of Chungnam National University.

## Conflict of interest

The authors declare that the research was conducted in the absence of any commercial or financial relationships that could be construed as a potential conflict of interest.

## Publisher's note

All claims expressed in this article are solely those of the authors and do not necessarily represent those of their affiliated organizations, or those of the publisher, the editors and the reviewers. Any product that may be evaluated in this article, or claim that may be made by its manufacturer, is not guaranteed or endorsed by the publisher.
